# Longitudinal evaluation of hippocampal subfields volumes and episodic memory in breast cancer patients and healthy controls

**DOI:** 10.1007/s11682-026-01162-6

**Published:** 2026-05-13

**Authors:** Baptiste Lerosier, Shailendra Segobin, Djelila Allouache, Sabine Noal, Christelle Levy, Ali R. Khan, Bradley G. Karat, Francois Christy, Florence Joly, Gael Chetelat, Francis Eustache, Bénédicte Giffard, Joy Perrier

**Affiliations:** 1 Université de Caen Normandie, Inserm, EPHE-PSL, Université Paris, CHU de Caen, GIP Cyceron, U1077, Neuropsychologie et Imagerie de la Mémoire Humaine (NIMH), Caen, 14000 France; 2 Medical Oncology Department, University Hospital of Caen, Caen, France; 3 Breast Committee Department, Centre François Baclesse, Caen, France; 4 Robarts Research Institute, Schulich School of Medicine and Dentistry, The University of Western Ontario, London, Canada; 5 Western Institute for Neuroscience, The University of Western Ontario, London, Canada; 6 Department of Medical Biophysics, Schulich School of Medicine and Dentistry, The University of Western Ontario, London, Canada; 7 Clinical Research Department, Centre François Baclesse, Caen, France; 8 Université de Caen Normandie, INSERM, ANTICIPE, Caen, France; 9 University of Caen Normandie, Services unit PLATON, Cancer and Cognition Platform, Caen, France; 10 Université de Caen Normandie, INSERM, U1237, Physiopathology and Imaging of Neurological Disorders (PhIND), Neuropresage Team, GIP Cyceron, Caen, France

**Keywords:** Breast neoplasms, Chemotherapy, Hippocampus, Retrieval, High-resolution MRI

## Abstract

Despite previous reports of hippocampal alterations in breast cancer patients, the structural evolution of hippocampal subfields in relationship with memory remains to be characterized in this population. We aimed to measure hippocampal subfield volumes and their links with memory performances before and after chemotherapy in breast cancer patients compared to healthy controls. Forty-two middle aged women were evaluated, including 19 patients assessed before (T1), one-month (T2) and one-year (T3) after chemotherapy, and 23 controls assessed at T1 and T3. Using high-resolution MRI, hippocampal subfields were automatically segmented. Derived volumetric measures were compared between groups at T1 and T3 and within the patient group (T1, T2, T3). Both encoding and retrieval memory performances were measured using a dedicated task. At T1 and globally (i.e., T1 and T3 together), patients showed lower retrieval performance and larger subiculum and whole hippocampal volumes compared to controls. Volumes of the whole hippocampus and of the Cornu Ammonis (CA) 4-Dentate gyrus increased over time in controls. Longitudinal comparisons within the patients’ group did not yield significant changes. Volumes were negatively associated with age in controls, and positively with education level in patients. Our results highlight structural differences in the subiculum and whole hippocampus of breast cancer patients both post-surgery and over the adjuvant treatment course. These modifications may reflect a combination of cancer-related and early perioperative factors, while the lack of subsequent longitudinal changes in patients prevents conclusions regarding the specific impact of chemotherapy. Additional studies with larger sample sizes will be of help to further our findings.

**Supplementary Information:**

The online version contains supplementary material available at 10.1007/s11682-026-01162-6.

## Introduction

Cancer and its treatments – particularly chemotherapy – have been associated with both cerebral alterations (Feng et al., 2019; McDonald, 2021; Zhou et al., 2022) and cognitive impairments, especially in memory functions (Amani et al., 2024; Lange et al., 2019; Zhou et al., 2024). Breast cancer (BC), given its high prevalence among women, is of particular concern. While therapeutic advances have significantly improved survival rates (Anampa et al., 2015), chemotherapy is still linked to neurotoxicity, primarily demonstrated in rodent studies (Taillibert et al., 2016). In humans, cross-sectional studies highlight a particular vulnerability of the hippocampus to chemotherapy (Amidi & Wu, 2019; Bergouignan et al., 2011; Dietrich et al., 2006; Kesler et al., 2013; Seigers et al., 2010). Moreover, human studies, although scarce, have shown both brain structure and function are modified before the start of chemotherapy (see Sousa et al., 2020, for review). Results from a human study have shown that elevated cytokines were related to hippocampal volume and memory performance before chemotherapy (Kesler et al., 2013). Moreover, animal studies have shown that tumor development altered hippocampal-related memory functioning (Winocur et al., 2018). These results thus suggest that inflammation, which may be the result of cancer biology and/or of stress-related processes, canlead to reduced hippocampal volume and memory impairments independently of chemotherapy. Given the hippocampus’s central role in memory encoding and retrieval (Bird & Burgess, 2008; Dolan & Fletcher, 1999), such structural changes could underlie the observed cognitive deficits. Yet, longitudinal studies in BC patients remain scarce, and none have specifically examined changes within hippocampal subfields, each of which may contribute differentially to memory processes.

Preclinical research shows that common chemotherapeutic agents reduce neurogenesis and increase cell death, particularly in the dentate gyrus (Sekeres et al., 2021), potentially leading to post-treatment cognitive impairments (Was et al., 2022). In humans, hippocampal deformation and reduced episodic memory performance have been observed in BC survivors post chemotherapy (Apple et al., 2017). The review by Peukert and colleagues (Peukert et al., 2020) indicated that seven studies have been published on the topic of hippocampal structure along with cognitive assessments, with most studies being cross-sectional and conducted after the end of treatment. Such cross-sectional designs prevent us from disentangling the effects of cancer from those of treatments. Moreover, while some studies have reported reduced hippocampal volume and neurogenesis after chemotherapy, others suggest chemotherapy-induced neuroinflammation may increase hippocampal volume (Schroyen et al., 2021), further complicating interpretations. Interestingly, tumor-bearing mice without chemotherapy also exhibit cognitive impairments and elevated pro-inflammatory cytokines, suggesting that cancer itself can affect brain function (Vardy et al., 2007; Winocur et al., 2018). In humans, studies suggest that cytokines, which can cross the blood-brain barrier and alter brain structure, may disrupt cognitive function and contribute to cognitive decline (Chen et al., 2021; Gibson & Monje, 2019; Sleurs et al., 2016; Tyagi et al., 2023). Only a few studies have focused on brain differences between BC patients and HCs at baseline, i.e., before the start of adjuvant treatments. Results support the presence of both brain structure and function alterations (McDonald et al., 2013; Scherling et al., 2011, 2012; Sousa et al., 2020); however, none of these studies have specifically focused on hippocampal subfields. Thus, the distinct contributions of cancer and chemotherapy to hippocampal structure changes remain unclear (Peukert et al., 2020), calling for further longitudinal studies on this topic.

Importantly, the hippocampus is not a uniform structure. Subfields such as Cornu Ammonis (CA) 1, CA3, the dentate gyrus, and the subiculum support different aspects of memory. CA1 and the subiculum are involved in retrieval, while CA2-3 and the dentate gyrus play a role in encoding (Eldridge et al., 2005; Suthana et al., 2011; Zeineh et al., 2003). The dentate gyrus is also the primary site of adult neurogenesis (Kempermann et al., 2015). Adult neurogenesis, which likely plays a role in cognitive processes, may be linked to the necessity for neuroplastic changes in individuals’ brains in reaction to environmental stimuli. The CA4, a relatively smaller hippocampal subfield, has received comparatively less attention in literature, partly due to the challenges associated with its segmentation. Despite their functional importance in several memory processes, information linking hippocampal subfield volumes with memory performance is lacking in BC patients.

The aim of the study was to longitudinally measure the volumetric evolution of hippocampal subfields in BC patients compared to HCs both before and after chemotherapy treatment. The second aim was to quantify the link between such modifications and episodic memory performance. A notable strength of our protocol is the inclusion of this thorough follow-up, encompassing the initial and longitudinal assessment of hippocampal subfields’ structure using high-resolution MRI, along with a detailed evaluation of both encoding and retrieval processes of memory using a dedicated task. This approach is anticipated to enhance the comprehension of the effects of peripheral phenomena (such as cancer and chemotherapy) on brain structure.

## Materials & methods

### Participants and study design

This study included twenty-five women with non-metastatic breast cancer. Among them, six were excluded as they did not participate in all MRI sessions scheduled in the protocol. The final patient group consisted of 19 BC patients. Patient inclusion criteria were (i) at least forty-five years old, (ii) no metastatic breast cancer, (iii) already undergone surgical treatment for cancer (tumorectomy or mastectomy) and scheduled to receive chemotherapy (fluorouracil, epirubicin, cyclophosphamide and docetaxel), followed by radiotherapy and/or hormone therapy if necessary (iv) no major psychiatric disorder according to DSM-IV criteria (Mini-International Neuropsychiatric Interview) before breast cancer diagnosis, (v) no neurological disease, (vi) no drug use or alcohol abuse, and (vii) no overall cognitive impairment according to the criteria of the Mini-Mental Status Examination.

Twenty-nine healthy control women (HCs), matched for age and education level, were included in the study. Six subjects did not complete all the MRI procedures required in the protocol. The final group of HCs was composed of twenty-three subjects. Inclusion criteria for control subjects were the same as for patients, except for those relating to cancer. All participants provided written informed consent prior to their inclusion in the study. All procedures were conducted in full accordance with the Declaration of Helsinki. The study was approved by the local ethics committee (CPP Nord Ouest III; n°ID-RCB: 2010-A00296-33).

The pretreatment assessment was conducted at least one month post-surgery but before the initiation of any adjuvant therapy (T1). Subsequent follow-up assessments were administered one month (T2) and one year (T3) following the end of chemotherapy for the patient cohort. Assessments for the control group were only carried out at intervals T1 and T3, aligning with the assessment schedule of the patients, with approximately 18 months between T1 and T3.

### Neuroimaging assessments and hippocampal subfields segmentation

Neuroimaging data were acquired using a 3T magnetic resonance imaging scanner (Intera Achieva 3T Quasar Dual camera, Philips Medical System) at Cyceron, a biomedical imaging center located in Caen, France. A morphological T1-weighted sequence was acquired for each participant (three-dimensional T1 fast field echo; sagittal; matrix size: 256 × 256 voxels with 180 contiguous sections; repetition time = 20 ms, echo time = 4.6 ms, flip angle = 20°, field of view = 256 × 256 mm², and slice thickness = 1 mm). Then, a high-resolution proton density-weighted sequence was acquired perpendicular to the long axis of the hippocampus (TR = 3,500 ms; TE = 19 ms; flip angle = 90°; 13 slices; slice thickness = 2 mm; interslice gap = 2 mm; in-plane resolution = 0.375 × 0.375 mm^2^, acquisition time = 7.4 min).

**Fig. 1:**
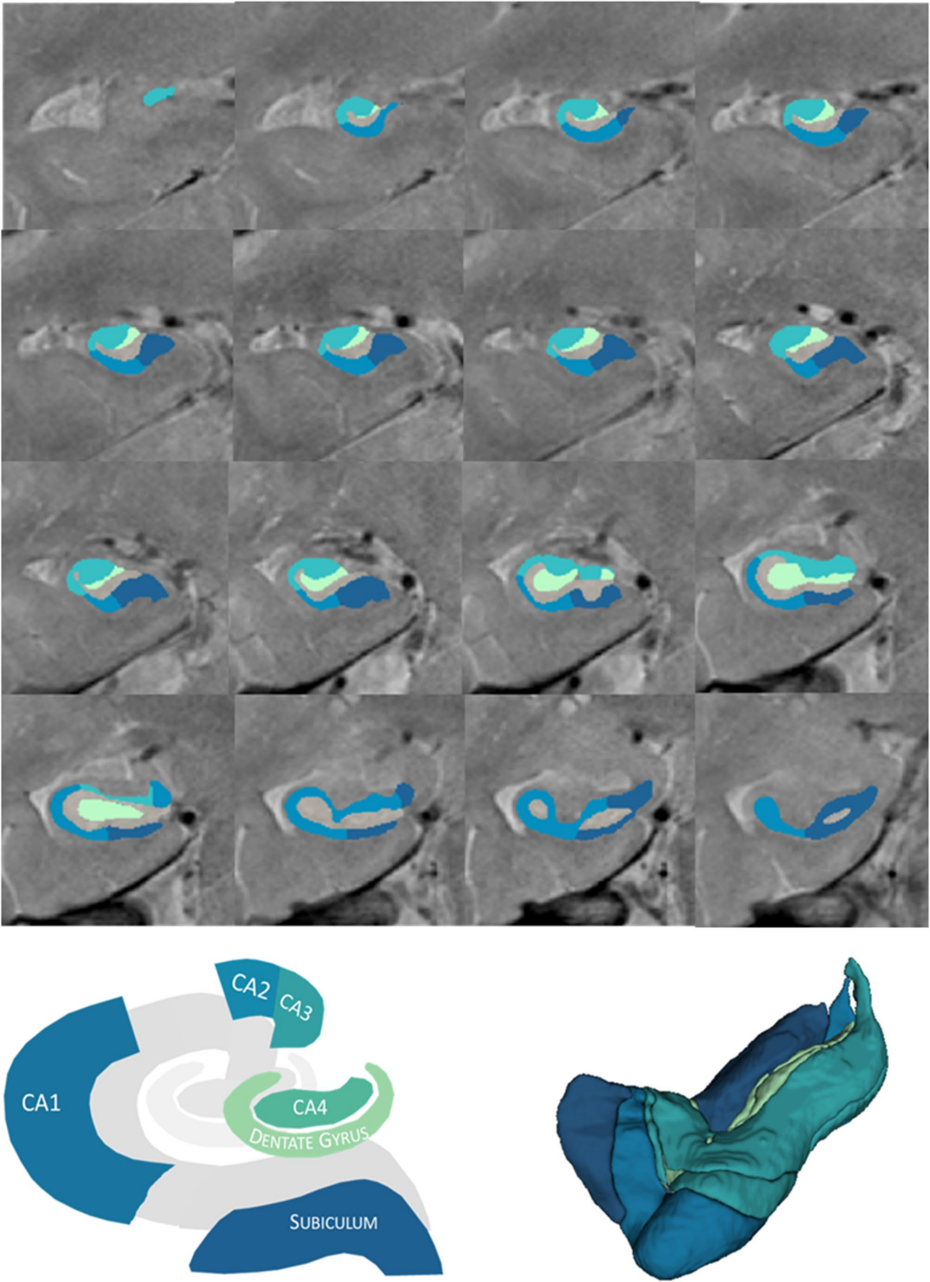
The upper panel shows coronal cross-sections of the hippocampal subfields segmented from high-resolution proton density–weighted images using the HippUnfold software. Dark blue: Subiculum; light blue: CA1; dark green: CA2–3; light green: CA4–DG. The lower panel illustrates the grouping of hippocampal subfields applied in the present study, following previous literature (Khlif et al., 2021; Ono et al., 2021; Zammit et al., 2017), with CA2 and CA3 merged into “CA2–3” and CA4 and DG combined into “CA4–DG” (left), as well as their corresponding 3D segmentation (right). CA, cornu ammonis; DG, dentate gyrus

Based on the high-resolution proton density-weighted images, the hippocampal subfields were segmented using version 1.3.3 of the HippUnfold software (DeKraker et al., 2022). See the supplemental materials for a detailed description of the method. Eight sub-regions were segmented by HippUnfold, namely CA1, CA2, CA3, CA4, Subiculum, SRLM, DG, and Cyst. According to previous methodologies investigating hippocampal subfield modifications associated with memory functioning (Khlif et al., 2021; Ono et al., 2021; Zammit et al., 2017), the CA2 and CA3 were combined into “CA2-3” and CA4 and the DG were merged into “CA4-DG” (Fig. 1). The SRLM and Cyst regions were not used in this study. Subfield volumes were measured in cubic millimeters (mm^3^).

### Anxiety assessment

Anxiety was measured with the State-Trait Anxiety Inventory, which is widely used to measure state and trait symptoms of anxiety. The first 20 items assess state anxiety (STAI-A), or how the participant feels right now; the second 20 items assess trait anxiety (STAI-B), or how the participant generally feels. Scores for the state and trait versions were reported separately, with higher values indicating higher anxiety symptoms.

### Episodic memory assessments

Assessments of both encoding and retrieval episodic memory processes were obtained from all participants at each time point of evaluation using a task developed in our laboratory, the Encoding, Storage, Retrieval task (ESR, Eustache et al., 1998). The ESR task is described in detail in Chetelat, 2003 and summarized in Fouquet et al., 2012. Briefly, two learning phases (superficial and deep) are performed with two separate lists of words from semantic categories and are followed by two distinct retrieval phases. Each encoding and retrieval phase is detailed in the supplemental materials. We here considered the main encoding and retrieval scores that were standardized into z-scores using the means and standard deviations derived from the control group’s scores at T1.

### Statistical analysis

Demographic and clinical characteristics of the sample were described using the mean, the standard deviation (SD), and the range for continuous data, and with frequencies and percentages for categorical data. Student’s t-tests were used to compare age, education level, and anxiety between groups at baseline (i.e., T1) when the normality assumption held true. Alternatively, the Mann-Whitney test was employed.

We first compared episodic memory performances and hippocampal subfield volumes between BC patients and HCs at T1, i.e., after surgery and before the start of chemotherapy.

Second, we used Linear Mixed Models (LMMs) to compare the evolution of both episodic memory performances and hippocampal subfield volumes across sessions. Models were fitted using the lme4 package. The full model specification included the subfield volume or memory score as the dependent variable, with session (categorical), group (categorical), and their interaction as fixed effects. Standardized continuous covariates (z-scores) for age and education level were included in all models, while estimated total intracranial volume (eTIV) was additionally included as a covariate when assessing subfield volumes. To account for repeated measures and intra-individual variability, a random intercept for each subject (1 | subject) was included. Models were estimated using Restricted Maximum Likelihood (REML), which inherently handles missing data assuming they are Missing At Random (MAR), thus retaining participants without requiring listwise deletion.

To assess the specific effects of chemotherapy on hippocampal subfield volumes across the cancer trajectory in BC patients only, similar mixed models were computed using data from the three time points (T1, T2, and T3), with session coded as a categorical fixed effect. Each subfield was analyzed separately.

Post-hoc pairwise comparisons for the mixed models were performed by extracting Estimated Marginal Means (EMMs) via the emmeans package. To strictly control for multiple comparisons across the numerous subfields and models, all post-hoc p-values were adjusted using the False Discovery Rate (FDR, Benjamini-Hochberg procedure).

Correlograms using Spearman’s correlations were computed to explore the relationships between variables of interest, i.e., demographical characteristics, memory scores at T1 and T3, and subfield volumes at T1. To account for the extensive number of tests in the correlation matrices, all resulting p-values were systematically adjusted using the False Discovery Rate (FDR) method. Statistical analyses were performed in RStudio version 2024.12.0.

## Results

### Participants’ characteristics

A total of 42 participants were analyzed. Baseline demographic information for both groups, as well as clinical characteristics of patients, is presented in Table 1. There were no significant differences in age, years of education, state anxiety, and trait anxiety between the groups. Most of the patients had undergone a tumorectomy, and cancer grades ranged from I to III. 

**Table 1:** Demographic characteristics of both groups and clinical characteristics of patients at baseline (T1)

	HCs		BC patients		HCs vs BC patients	
	*n* = 23		*n* = 19			
	Mean (SD)	Range	Mean (SD)	Range	t/ U	*p*-value
Age	56.09 (7.75)	44 - 68	53.95 (4.88)	44 - 61	1.04	0.30^a^
Years of education	12.30 (2.91)	7 - 17	11.32 (3.35)	7 - 17	1.02	0.31^a^
State anxiety	28.04 (5.38)	20 - 41	32.05 (9.06)	22 - 50	163	0.16^b^
Trait anxiety	36.65 (9.44)	20 -51	36.58 (9.08)	23 - 60	0.03	0.98^a^
Type of surgery, n (%)						
Mastectomy			3 (16%)			
Tumorectomy			16 (84%)			
Severity of breast cancer, n (%)						
I			4 (21%)			
II			8 (42%)			
III			7 (37%)			
Menopausal status, n (%)						
Premenopausal	Non available		9 (47%)			
Postmenopausal			10 (53%)			

*p*-values were calculated using a T-test when the normality hypothesis was true (^a^); otherwise, Mann-Whitney test was applied (^b^). State anxiety and Trait anxiety were assessed using STAI-A and STAI-B (State-Trait Anxiety Inventory), respectively. *n *number of subjects, *SD* Standard deviation, *HCs* healthy controls, *BC* breast cancer

**Table 2:** Description of memory score and hippocampal subfields in HCs and breast cancer patients at each time points

		HCs		BC patients	
		Mean (SD)	Range	Mean (SD)	Range
Encoding Score	T1	0.00 (1.00)	-2.01 – 1.41	-0.42 (1.00)	-2.58 – 1.41
	T2	NA		0.06 (1.22)	-3.72 – 1.41
	T3	0.01 (0.94)	-2.01 – 1.41	-0.12 (1.28)	-2.58 – 1.41
Retrieval Score	T1	0.00 (1.00)	-2.07 – 1.47	-0.92 (1.32)	-3.08 – 1.98
	T2	NA		-0.31 (1.26)	-2.07 – 2.49
	T3	0.26 (1.19)	-3.08 – 1.98	-0.47 (1.02)	-2.07 – 1.98
Whole Hippocampus volume mm^3^	T1	2341 (339)	1779 - 2888	2534 (254)	2127 - 3023
	T2	NA		2553 (241)	2164 - 2983
	T3	2445 (260)	1916 - 2907	2544 (235)	2133 - 2914
Subiculum volume mm^3^	T1	458 (75)	294 - 642	500 (65)	344 - 654
	T2			506 (73)	360 - 680
	T3	480 (59)	343 - 628	507 (66)	386 - 667
CA1 volume mm^3^	T1	697 (145)	345 - 1008	757 (117)	508 - 970
	T2	NA		763 (95)	590 - 982
	T3	726 (144)	362 - 982	758 (88)	607 - 922
CA2-3 volume mm^3^	T1	354 (61)	252 - 513	370 (55)	272 - 490
	T2	NA		369 (57)	271 - 539
	T3	368 (63)	252 - 558	365 (57)	270 - 487
CA4-DG volume mm^3^	T1	280 (67)	118 - 403	307 (63)	176 - 456
	T2	NA		306 (51)	203 - 408
	T3	298 (58)	157 - 408	306 (65)	215 - 449

*CA* cornu ammonis, *DG* dentate gyrus, *HCs *healthy controls, *BC* breast cancer, *T1* after surgery and before chemotherapy, *T2* one month after the end of chemotherapy, *T3 *one year after the end of chemotherapy

### Comparison of both episodic memory performance and hippocampal subfield volumes between HCs and BC patients at T1

At T1, after controlling for age, education level, and estimated intracranial volume (eTIV), patients had significantly lower episodic memory retrieval scores (*F* = 4.93, *p* = 0.033) compared to HCs. Regarding volumes, there was a trend toward larger volumes in BC patients for the subiculum (*F* = 3.96, *p* = 0.054) and the whole hippocampus (*F* = 4.04, *p* = 0.052). Other hippocampal subfield volumes did not significantly differ between groups (CA1: *p* = 0.19; CA2-3: *p* = 0.38; CA4-DG: *p* = 0.12). See Table 2 for means and standard deviations.

**Fig. 2:**
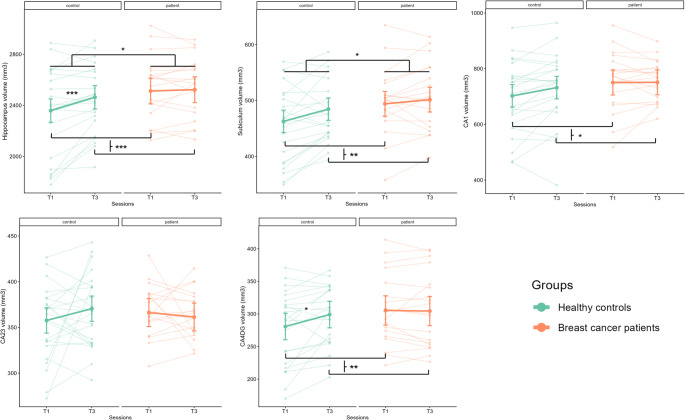
Longitudinal trajectories of hippocampal subfield volumes in Healthy Controls (HCs) and Breast Cancer (BC) patients. Transparent lines and points represent individual raw trajectories (spaghetti plots) showing within-subject changes from baseline (T1) to follow-up (T3). Solid lines and larger points represent the Estimated Marginal Means (EMMs) derived from the linear mixed-effects models. Error bars indicate the 95% confidence intervals of the EMMs

### Evolution of both episodic memory performance and hippocampal subfield volumes between HCs and BC patients between T1 and T3

The longitudinal analysis of encoding and retrieval memory scores showed a significant effect of group for the retrieval score (*F* = 5.957, *p* = 0.02), patients had lower z-scores than HCs, suggesting relatively poorer performance in memory retrieval over the cancer trajectory (*d* = 0.730, 95% CI = [-1.252 – -0.137], *p* = 0.02). The group-by-session interaction was not significant.

Significant session effects were found for the whole hippocampus (*p* < 0.001), subiculum (*p* = 0.002), CA1 (*p* = 0.013), and CA4-DG (*p* = 0.002), with generally increased volumes from T1 to T3. Significant main group effects were found for the whole hippocampus (*p* = 0.032) and the subiculum (*p* = 0.045), with larger volumes in BC patients than in HCs. Finally, significant group-by-session interactions were found for the whole hippocampus (*p* = 0.014) and CA4-DG (*p* = 0.023). Post-hoc analyzes revealed a significant increase in volumes from T1 to T3 in HCs (*p* < 0.001 and *p* = 0.01, respectively; FDR-corrected *p* < 0.001 and *p* = 0.011), whereas no significant changes were observed in BC patients (*p* = 0.98 and *p* = 0.99, respectively; FDR-corrected *p* = 0.703 and *p* = 0.878). To directly visualize these longitudinal trajectories and interindividual variability, spaghetti plots displaying individual-level connecting lines along with the superimposed Estimated Marginal Means (EMMs) and their confidence intervals are presented in Fig. 2; Table 3.

**Table 3:** Statistical results following the comparisons of the evolution across sessions T1 and T3 of hippocampal subfield volumes in HCs and in BC patients

Hippocampal subfields	*R*² of the mixed model	Fixed effects	Estimates	Standard errors	df	*t* values	95% Confidence intervals	*p*-values
Whole hippocampus	0.92	session	104.42	24.68	40	4.23	[56.0, 152.79]	< 0.001***
		group	153.65	69.31	42.56	2.22	[17.81, 289.48]	0.032*
		group*session	-94.01	36.69	40	-2.56	[-165.93, -22.09]	0.014*†
Subiculum	0.87	session	21.85	6.74	40	3.24	[8.64, 35.06]	0.002**
		group	31.35	15.18	45.97	2.07	[1.59, 61.10]	0.045*
		group*session	-14.41	10.02	40	-1.44	[-34.05, 5.23]	0.15
CA1	0.89	session	29.70	11.42	40	2.60	[7.31, 52.09]	0.013*
		group	47.82	30.75	43.09	1.56	[-12.44, 108.08]	0.13
		group*session	-28.79	16.98	40	-1.70	[-62.08, 4.49]	0.098
CA2-3	0.31	session	12.88	8.85	40	1.46	[-4.46, 30.22]	0.15
		group	8.61	10.51	72.27	0.82	[-11.98, 29.21]	0.42
		group*session	-17.76	13.15	40	-1.35	[-43.54, 8.03]	0.18
CA4-DG	0.89	session	18.32	5.49	40	3.34	[7.56, 29.08]	0.002**
		group	24.62	15.50	42.50	1.59	[-5.75, 54.97]	0.12
		group*session	-19.25	8.16	40	-2.36	[-35.24, -3.26]	0.023*†

Comparisons were made between groups (i.e., healthy controls and breast cancer patients) and across sessions, considering before (T1) and one-year (T3) after the end of chemotherapy for patients and at similar time points in HCs. Each subfield corresponds to an independent mixed model with age, education level and TIV as covariates. CA = cornu ammonis; DG = dentate gyrus. R², conditional R² corresponding to both fixed and random effects. Results are reported following best practices (Meteyard & Davies, 2020). *P*-values presented in this table are uncorrected values for the mixed-model fixed effects (* *p* < 0.05, ** *p* < 0.01, *** *p* < 0.001). A dagger symbol (†) next to an interaction term indicates that the subsequent post-hoc tests designed to decompose this interaction were significant after False Discovery Rate (FDR) correction

### Volumetric evolution in hippocampal subfields among BC patients along the cancer trajectory

Mixed-model analyses throughout the eighteen-month follow-up period within the patient group did not reveal any significant differences, neither linear nor quadratic (all *p*s > 0.05, see Table 4). Hippocampal subfield volumes thus remained stable throughout the entire cancer trajectory when considering only the group of patients.

**Table 4:** Evolution of hippocampal subfield volumes in breast cancer patients across the three sessions of evaluations

Hippocampal subfields	Linear session effects		Quadratic session effects	
	β	*p*-value	β	*p*-value
Subiculum	5.26	0.38	-2.16	0.72
CA1	0.64	0.95	-4.78	0.63
CA2-3	-3.45	0.70	-1.25	0.89
CA4/DG	-0.66	0.91	0.41	0.95

Each line in the table corresponds to an independent mixed model. *CA* cornu ammonis, *DG* dentate gyrus

### Relationship between variables of interest at T1 both in HCs and BC patients

**Fig. 3:**
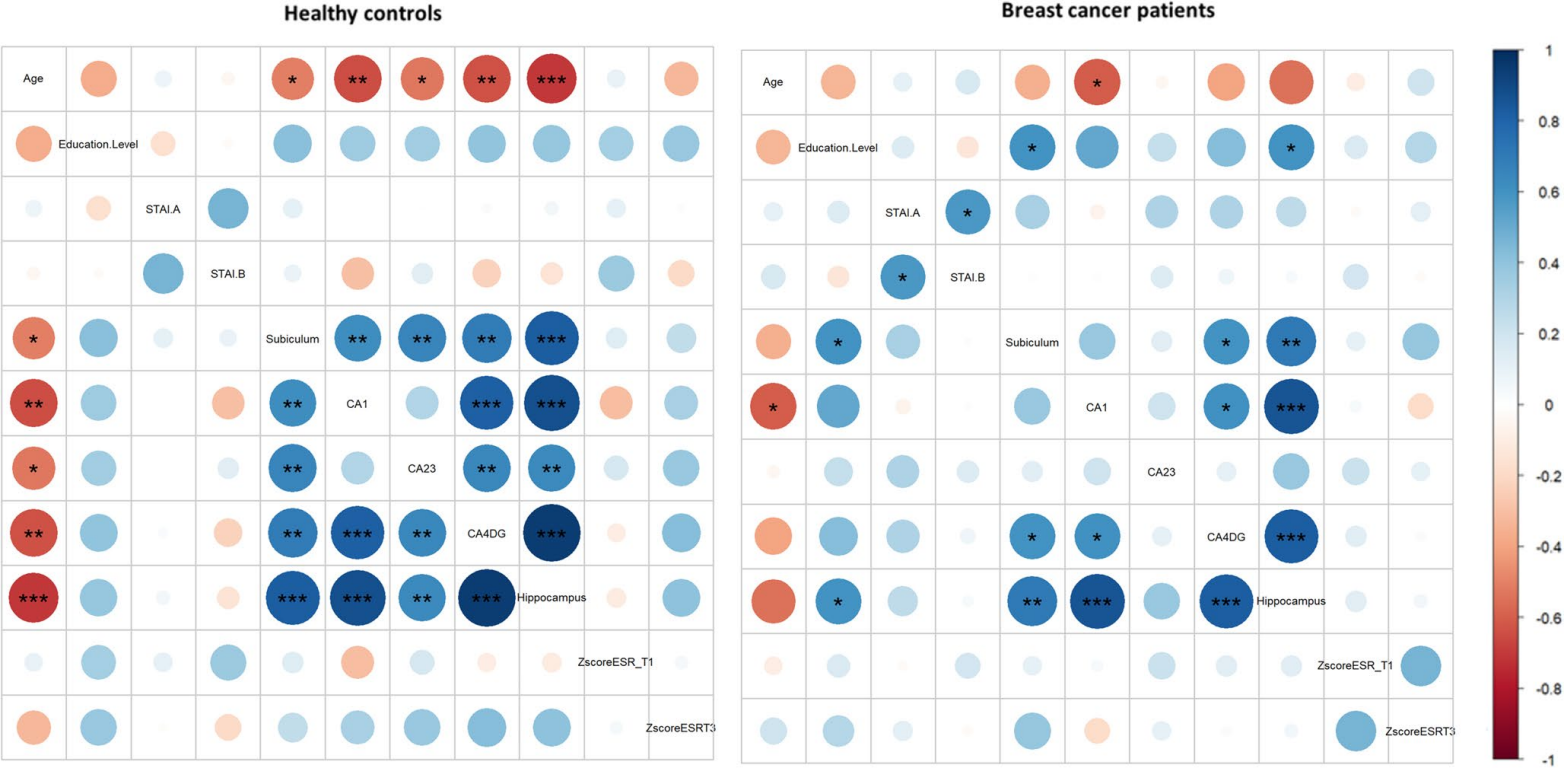
Correlograms of Spearman’s correlations between hippocampal subfields’ volume and variables of interest, including age, education level and memory scores for each group, healthy controls (left) and breast cancer patients (right). Blue and red circular diagram represent positive and negative correlations, respectively. The strength of the relation is indicated by the degree of filling of the circle, the more the circle is filled, the higher is the r value. We considered significant *p* values at 0.05 with *, *p* < 0.05; **, *p* < 0.01 and ***, *p* < 0.001. ESR, Encoding, Storing, Retrieval

After applying FDR correction for multiple comparisons, correlograms revealed significant negative relationships between age and hippocampal volumes at T1 in the HCs group. Older age was associated with lower volumes. In BC patients only, education level was positively associated with hippocampal volumes, with higher education levels associated with larger volumes Fig. 3.

## Discussion

The current study aimed to investigate the evolution of memory performance and hippocampal subfield volumes from before chemotherapy (i.e., after surgery, T1) to one year later in BC patients compared to HCs. Consistent with previous findings (Lange et al., 2019; Perrier et al., 2020), while episodic memory performance was overall lower in patients than in controls, no further decline was observed one year after chemotherapy. This result aligns with the concept of Cancer-Related Cognitive Impairment (CRCI) and suggests, in the current sample of patients, deleterious effects of cancer on memory processes. Regarding hippocampal subfield volumes, results showed a trend toward larger subiculum volume in patients before chemotherapy, indicating that cancer-related processes (i.e., either psychological or biological) may contribute to brain changes, notably through neuroinflammation processes. Longitudinal evaluation within the patient group (i.e., T1, T2, T3), revealed no significant volume changes over time. The potential deleterious effects of chemotherapy and subsequent adjuvant therapies cannot be ruled out, given that HCs had an increase in CA4-DG volume from T1 to T3, which was not observed in BC patients. These findings suggest that, in the current sample, the overall therapeutic management may hinder the increase in hippocampal volume in BC patients, particularly in the CA4-DG subfield.

Although unexpected, the lack of hippocampal volume reduction post-chemotherapy reported in the current sample aligns with two prior studies, which also reported no such effect (Inagaki et al., 2007; Yoshikawa et al., 2005). These studies compared BC patients receiving or not chemotherapy and evaluated them either 1 year or at least 3 years after diagnosis. The authors did not find any significant difference in hippocampal volume between groups. As a side note, neither of these studies was longitudinal, nor did they include an HC group. Nonetheless, chemotherapy has been associated with neurotoxic effects, including, but not limited to, the suppression of hippocampal neurogenesis, neuroinflammation, increased oxidative stress, and cell death (Dahiya et al., 2023), potentially leading to hippocampal atrophy (Amidi & Wu, 2019; Bergouignan et al., 2011; Kesler et al., 2013; Peukert et al., 2020). In the current study, the lack of volume increase over time in CA4-DG among patients may reflect impaired neurogenesis, as the dentate gyrus (DG) is the main source of new neuron formation. Although not directly measured here, this is consistent with existing hypotheses in the field.

Most prior studies reporting chemotherapy-related atrophy were cross-sectional and conducted well after treatment. Such designs make it difficult to distinguish cancer effects from treatment ones. Cancer alone can alter hippocampal structure before treatment onset (Chen et al., 2024), likely via neuroinflammatory mechanisms due to the tumor (Patel et al., 2015; Schroyen et al., 2021). Neuroinflammation of hippocampal tissue is characterized by an excessive production of interleukin-6. This process could begin with an increase in volume before causing long-term damage to the tissue and resulting in volume reduction (Daniel et al., 2024; Marsland et al., 2008; Satizabal et al., 2012). Previous reports have, indeed, revealed both brain structure and function alterations before chemotherapy (Cimprich et al., 2010; Scherling et al., 2012). In the current study, the trend toward increased subiculum volume at T1 in BC patients may reflect such early neuroinflammation, which aligns with previous findings in animals (Chen et al., 2024). This result still concurs with another human study reporting no pre-treatment differences in hippocampal volume between BC patients and HCs (McDonald et al., 2010). Such a discrepancy may stem from methodological factors – lower MRI resolution, smaller sample size, higher education level of patients – suggesting that cancer-related changes might be subtle and/or limited to specific subgroups, as previously proposed (McDonald et al., 2013).

Beyond cancer and chemotherapy, other confounding factors - such as cognitive reserve (Ahles et al., 2010), anxiety (Perrier et al., 2020), sleep (Mulholland et al., 2023; Rehel et al., 2024), and/or fatigue (Hampson et al., 2015; Menning et al., 2015), but also clinical characteristics including days since surgery and cancer grade (McDonald et al., 2010) – might influence hippocampal volume. In the current study, education level positively correlated with subfield volumes, potentially reflecting a protective effect of cognitive reserve in our sample of patients, as previously proposed (Ahles et al., 2010; Ahles & Hurria, 2018).

Regarding longitudinal evolution, we observed a main effect of session for most subfields, with volumes increasing over time, but this was primarily driven by HCs. Given that large longitudinal studies typically report stable or declining hippocampal volumes from midlife onward (Nobis et al., 2019), this apparent increase must be interpreted with high caution. This finding may be attributable to measurement variability across sessions, sampling effects, or unmeasured residual confounding. Consequently, and because within-patient analyses across T1, T2, and T3 did not show significant session effects, it is difficult to firmly attribute these divergent group trajectories to a chemotherapy-related effect based on the current data alone. This highlights the need for large-scale, longitudinal studies to rigorously control for both methodological biases and lifestyle confounders.

We expected hippocampal subfield volumes - especially the Subiculum and CA1, which are key to episodic memory retrieval – to be related to memory performance. However, no association was found between hippocampal subfield volumes and memory scores. While low statistical power may explain this result in patients, hippocampal volume alone might not account for memory impairments in BC patients. Indeed, memory retrieval involves not only the hippocampus, but also prefrontal regions that support control processes and executive functions essential for retrieval strategies (Torres-Morales & Cansino, 2024). Furthermore, Eyler et al. suggest that a simple model linking cognitive performance to brain or hippocampal volume may be inaccurate (Eyler et al., 2011). Combining structural and functional neuroimaging may better clarify this relationship.

This study has several limitations. First, the sample size was relatively modest, with approximately twenty participants per group. This may have limited the statistical power to detect subtle longitudinal or interaction effects, particularly when examining multiple hippocampal subfields and cognitive outcomes. This sample size reflects the stringent inclusion criteria applied to ensure a homogeneous clinical sample, notably with respect to age range and treatment regimens, as well as the demanding longitudinal design requiring repeated MRI and detailed cognitive assessments. Second, we were unable to evaluate the potential impact of anxiety in our sample, despite its well-documented influence on hippocampal volume. It cannot be ruled out that the absence of elevated anxiety levels in our patient group compared to HCs — potentially attributable to specific psychological traits associated with adherence to the demanding experimental protocol — may have limited the detection of additional hippocampal alterations. Third, our baseline assessment (T1) was conducted at least one month after surgery but before adjuvant treatments. Although previous studies have reported the presence of cognitive deficits prior to surgery (Lange et al., 2020), we still cannot rule out the possibility that perioperative factors may have contributed to the brain structure and memory differences observed at this time point, alongside the effects of the cancer itself. Another limitation of this study is the potential impact of test-retest effects on memory tests, where patients completed the test one more time than control subjects. This could increase their familiarity with the test and influence the results. However, in the present study, patients did not show better performance than controls on memory measures, suggesting that additional test exposure did not translate into improved outcomes. It is also important to recognize that certain unmeasured variables could influence our results. Thus, to enhance the validity of future studies, it would be prudent to incorporate additional variables and gather more exhaustive data, particularly relating to subjects’ lifestyles and quality of life.

By originally focusing on hippocampal subfields, and episodic memory, this study adds to the evidence that a combination of cancer-related and early perioperative factors may affect brain structure even before the start of adjuvant treatments. Notably, the current sample of BC patients exhibited larger subiculum and whole hippocampal volumes one month after surgery and prior to chemotherapy, which may result from neuroinflammation as previously proposed in animal studies. Further longitudinal studies controlling for additional confounding factors are needed to pursue these findings and clarify the associated mechanisms.

## Supplementary Information

Below is the link to the electronic supplementary material.

Supplementary Material 1 (DOCX 23.6 KB)

## Data Availability

The data that support the findings of this study are available from the last authors (BG and JP) upon reasonable request.
