# Supplementary material for: Longitudinal evaluation of hippocampal subfields volumes and episodic memory in breast cancer patients and healthy controls
Source: Brain Imaging Behav. 2026 May 13;20(3):94. doi: 10.1007/s11682-026-01162-6 (PMC13167817; doi:10.1007/s11682-026-01162-6)
Supplement: Supplementary file 1 — Supplementary Material 1 (DOCX 23.6 KB) [file 11682_2026_1162_MOESM1_ESM.docx]

# Longitudinal evaluation of hippocampal subfields volumes and episodic memory in breast cancer patients and matched healthy controls

Baptiste Lerosier ^1^, Shailendra Segobin ^1^, Djelila Allouache ^2,3^, Sabine Noal ^2,3^, Christelle Levy ^2,3^, Ali R. Khan ^4,5,6^, Bradley G. Karat ^4,5^, Francois Christy ^7^, Florence Joly ^2,7,8,9^, Gael Chetelat ^10^, Francis Eustache ^1^, Bénédicte Giffard ^1,9,11^, Joy Perrier PhD ^1,11^

^1^ Université de Caen Normandie, Inserm, EPHE-PSL, PSL Université Paris, CHU de Caen, GIP Cyceron, U1077, Neuropsychologie et Imagerie de la Mémoire Humaine (NIMH), Caen, 14000, France.

^2^ Medical Oncology Department, University Hospital of Caen, Caen, France.

^3^ Breast Committee Department, Centre François Baclesse, Caen, France.

^4^ Robarts Research Institute, Schulich School of Medicine and Dentistry, The University of Western Ontario, London, Canada.

^5^ Western Institute for Neuroscience, The University of Western Ontario, London, Canada.

^6^ Department of Medical Biophysics, Schulich School of Medicine and Dentistry, The University of Western Ontario, London, Canada.

^7^ Clinical Research Department, Centre François Baclesse, Caen, France.

^8^ Université de Caen Normandie, INSERM, ANTICIPE, Caen, France.

^9^ Cancer & Cognition, Platform, Ligue Nationale Contre le Cancer, Caen, France.

^10^ Université de Caen Normandie, INSERM, U1237, Physiopathology and Imaging of Neurological Disorders (PhIND), Neuropresage Team, GIP Cyceron, Caen, France.

^11^ These authors equally contributed to this work

**Corresponding Author:**

Joy Perrier

Neuropsychologie & Imagerie de la Mémoire Humaine

Unité de recherche 1077 INSERM-EPHE-UNICAEN, Pôle des Formations et de Recherche en Santé (PFRS), 2 rue des Rochambelles F-14032 Caen Cedex CS 14032, France

Phone: +33(0) 2 31 56 83 83; E-mail: [joy.perrier@unicaen.fr](mailto:joy.perrier@unicaen.fr)

Detailed description of the ESR memory task

During the first list, participants determined if the first and last letters of orally presented words were in alphabetical order without any instruction to memorize. At the end of this incidental superficial encoding phase, a recognition phase was carried out where patients had to recognize the 16 target words among distractors. Each target word was presented visually, one by one, with three distractors: one semantically linked, one phonetically linked, and the third with no link to the target word. For each of these 16 presentations, patients were systematically required to point to a word with their finger, the one they recognized, or otherwise the one they chose at random. For the second list, patients were asked to memorize the words. To induce semantic processing, they had to orally generate a sentence that defined or described the presented word. Every two words, an immediate cued recall task was performed using a semantic category cue to ensure encoding and reinforce its semantic nature. If the patient failed, they were reminded of the word and asked to create a sentence containing the target and recall it in response to its categorical cue. At the end of this 16-word intentional deep encoding, patients are asked to recall as many words as possible, in any order and without time limitation. Performance in recognition after incidental superficial encoding from the first list is assumed to mainly reflect encoding capacity, as recognition is supposed to compensate for potential retrieval deficits. In contrast, performance in free recall after intentional deep encoding is assumed to preferentially reflect retrieval capacity as encoding is supported, thereby compensating for potential deficits in spontaneous encoding capacities.

## Hippocampal subfield segmentation

The pipeline is designed for the segmentation and analysis of hippocampal subfields and MRI images undergo preprocessing and cropping to focus on hippocampi. A U-Net neural network architecture [36] is applied to segment various components, including hippocampal grey matter (GM), the stratum radiatum lacunosum and moleculare (SRLM), as well as surrounding hippocampal structures. Laplace's equation is solved across three dimensions thereby establishing a geodesic coordinate framework. Scattered interpolants are employed to establish equivalent coordinates between the native Cartesian space and the unfolded space. Unfolded surfaces with template subfield labels [37] are transformed into the native folded configurations specific to individual subjects. Morphological features such as thickness are extracted from these surfaces within the context of the native folded space. Finally, volumetric subfields are generated by labelling the hippocampal gray matter voxels using the unfolded coordinates and the subfield atlas defined on these coordinates. To ensure the reliability of the outcomes, quality control was conducted with an expert (BK) in hippocampal anatomy to assess the efficacy of HippUnfold in segmenting the sequence employed.

Relationship between hippocampal subfield volumes and retrieval episodic memory score at T1

A general linear model was used to test if the subfield volumes could explain the memory scores at T1. However, no association was found between hippocampal subfield volumes and episodic memory retrieval at T1.

Table S1 Association between hippocampal subfield volume and episodic memory retrieval at T1

| **Episodic Memory retrieval at T1** | F | p-value |
| --- | --- | --- |
| Subiculum Volume | 0.407 | 0.527 |
| Group* Subiculum volume | 0.167 | 0.685 |
| CA1 Volume | 2.299 | 0.138 |
| Group*CA1 volume | 0.341 | 0.563 |
| CA2-3 Volume | 1.501 | 0.228 |
| Group*CA2-3 volume | 0.397 | 0.533 |
| CA4-Dentate gyrus volume | 1.400 | 0.244 |
| Group*CA4-Dentate gyrus volume | 0.220 | 0.641 |

Note: CA = cornu ammonis

Relationship between hippocampal subfield volumes and variation of retrieval episodic memory score (T1-T3)

A general linear model was employed to assess whether subfield volumes in explaining the variability in episodic memory retrieval scores from T1 to T3. Nevertheless, no significant association was observed between hippocampal subfield volumes and episodic memory retrieval.

Table S2 Association between hippocampal subfield volume and variation of episodic memory retrieval.

| **Variation of Episodic memory retrieval between T1 and T3** | F | p-value |
| --- | --- | --- |
| Subiculum volume | 0.075 | 0.785 |
| Group* Subiculum volume | 0.410 | 0.526 |
| CA1 volume | 0.013 | 0.911 |
| Group*CA1 volume | 0.470 | 0.497 |
| CA2-3 volume | 0.026 | 0.874 |
| Group*CA2-3 volume | 0.148 | 0.702 |
| CA4-Dentate gyrus volume | 0.132 | 0.718 |
| Group*CA4-Dentate gyrus volume | 0.031 | 0.862 |

Note: CA = cornu ammonis
